# Fistula in Ano

**Published:** 1895-04

**Authors:** Joseph B. Bacon

**Affiliations:** Chicago


BACON, JOSEPH B., CHICAGO: FISTULA IN ANO. (*Northwestern Medical Journal.*) In the St. Mark's Hospital for Rectal Diseases, in London, the statistics show fistula to be the most frequent of all rectal diseases that apply for treatment, and that a large percentage of these applicants have been unsuccessfully operated upon by physicians, before applying to the hospital. The same condition would probably be found in this country, had we a great centre for collecting these patients, where accurate statistics could be tabulated.

There were many good reasons, before the antiseptic treatment of wounds, why so many fistulas could not be successfully handled, but the main reasons are:

First, the general practitioner has failed to realize that all fistulas are the result of abscesses, and that the fistulous tract is but the collapsed abscess wall, and that naturally there would be a tendency for the pus to have burrowed, in most cases, in various directions, leaving this collapsed abscess wall complicated by numerous channels, radiating from the central canal.

Second, that until recently the student was not required to make careful dissections of the pelvic fascias, perineal and anal regions, thus leaving him, when in practice, with a vague idea of the direction the pus, from an abscess in these regions, would likely take in accordance with the anatomical arrangements of the tissues. Repeated dissections, only, will give one an idea of the possibilities of the complications that may attend these abscesses, and also show how difficult the diagnosis and treatment may become.

Usually, the abscesses will occur either subcutaneously, submucously, or in the ischio-rectal fossæ, and still rarer, yet most difficult, when it occurs in the superior peri-rectal space. The various types of resulting fistulas will depend upon where and in how many places these abscesses have gained an outlet. While the division of some authors into first, blind internal, complete, and incomplete; second, blind external, complete, and incomplete; third, complete internal; fourth, complete external, will describe the greater number of fistulas one meets, there may be various complications with those that would make a more extensive classification possible.

The etiology of abscesses here may be the same as in other locations. Likewise, the symptoms do not differ from any other source of irritation around the anal region, except when there is an outlet for the pus secretion, in such a location as to attract attention from its constant escape.

There are no secret remedies for the treatment of fistulæ. The same surgical principles apply here, as well as elsewhere. Absolute cleanliness, and disinfection of the tract with peroxide of hydrogen, listerine, bichloride solutions, and the various antiseptics, together with keeping the fistulous tract patulous, will cure a great many of these cases. Allingham's method of keeping the external opening dilated, is an excellent one, and simply consists in inserting into the opening a bone collar button that has had a hole drilled through it lengthwise. Curetting, or cauterizing the tract sufficiently to set up inflammatory conditions, will hasten granulation and repair.

These palliative measures should only be used for a short time, when, if the tract has not healed, radical measures for laying open the tract must be taken, for long-continued suppuration always tends to amyloid degeneration of the viscera, with general constitutional disturbances.

In doing a radical operation for fistula, there are some important points to be observed:

1. Never sever the sphincters at more than one place at the same operation, no matter what the complications may be, otherwise incontinence is sure to follow.

2. Unless all the channels are followed up and laid open, the operation will fail of its purpose.

3. Fistula, resulting from tubercular abscess, must not be operated upon, if there is sufficient tissue destruction of lung to produce hectic fever, sweats, etc., unless the fistula is causing severe painful spasms of the sphincters, then it should be divided at any stage.

4. After laying the fistulous tract open, the wound most be made to heal from the bottom, and as the cutaneous or mucous side of the wound is better nourished, it will throw out a more healthy granulation, that tends to bridge over and close the slower granular surface at the bottom, thus leaving a fistula remaining.

5. When the fistulous tract is not too complicated, it should be dissected out entire, and the wound brought together, beginning at the bottom, with continuous catgut sutures, and approximating the surfaces in successive layers, until the whole wound is closed.

All operations about the anus, although theoretically septic, should be done with the same care, and dressed the same as for a laparotomy, then more of them will heal without formation of pus.

The general prejudice among the laity against having fistulæ cured, has been taught them by ignorant physicians, and to disabuse their minds of this idea will take time and concerted action of the profession. It is a common thing to see patients in the hospitals, whose viscera have undergone amyloid degeneration, and they have become hopelessly wrecked by a long-continued septic absorption from a fistulous tract, that a minor operation would have relieved at its commencement.

